# A novel compound DBZ alleviates chronic inflammatory pain and anxiety-like behaviors by targeting the JAK2-STAT3 signaling pathway

**DOI:** 10.1016/j.jbc.2025.110223

**Published:** 2025-05-09

**Authors:** Bao Sun, Mengyao Wu, Yilin Ru, Yaxi Meng, Xin Zhang, Fengyun Wang, Zhaodi Xia, Le Yang, Yufei Zhai, Gufeng Li, Jinming Hu, Bing Qi, Pu Jia, Sha Liao, Haiyan Lu, Minggao Zhao, Xiaohui Zheng

**Affiliations:** 1 College of Life Sciences, Key Laboratory of Resource Biology and Biotechnology in Western China, Ministry of Education, College of Life Science, Northwest University, Xi’an, PR, China; 2 Department of Pharmacy, The Second Affiliated Hospital of Xi'an Medical University, Xi'an, PR, China; 3 Department of Pharmacy, Xi'an Daxing Hospital, Xi'an, PR, China; 4 Precision Pharmacy & Drug Development Center, Department of Pharmacy, Tangdu Hospital, Fourth Military Medical University, Xi’an, PR, China

**Keywords:** chronic inflammatory pain, anxiety, DBZ, JAK2-STAT3, astrocytes

## Abstract

Chronic pain profoundly disrupts patients' daily lives and places a heavy burden on their families. Tanshinol Borneol Ester (DBZ), a novel synthetic derivative, has demonstrated anti-inflammatory and anti-atherosclerotic effects, yet its impact on the central nervous system remains largely unexplored. This study systematically examines the central nervous system effects of DBZ through a combination of *in vivo*, *in vitro*, network pharmacology, and molecular docking approaches. *In vivo*, we utilized a mouse model of chronic inflammation induced by complete Freund’s adjuvant to evaluate DBZ’s influence on pain, anxiety-like behaviors, and its modulation of inflammatory and oxidative stress markers within the anterior cingulate cortex. *In vitro* studies on primary mouse astrocytes assessed DBZ’s effects on cell viability and inflammatory marker expression. Network pharmacology was employed to elucidate DBZ’s potential molecular targets and pathways, while molecular docking provides valuable docking confirming its interactions with key components of the JAK2-STAT3 signaling pathway. Our findings demonstrate that DBZ effectively mitigates complete Freund’s adjuvant–induced chronic pain and anxiety-like behaviors. It significantly suppresses astrocytes activation, reduces levels of pro-inflammatory cytokines IL-1β, IL-6, and TNF-α, and diminishes oxidative stress markers such as reactive oxygen species and malondialdehyde, while enhancing superoxide dismutase levels. Moreover, DBZ modulates excitatory synaptic proteins and the JAK2-STAT3 signaling pathway in the anterior cingulate cortex, suggesting its role in neuroprotection. These results position DBZ as a promising candidate for the treatment of chronic pain and anxiety, offering a potential foundation for the development of new therapeutic agents.

Chronic pain is an important health problem in clinical practice, often leading to immune dysfunction, dietary pattern changes, cognitive impairment, and adverse stress responses. These effects contribute to insomnia, anxiety, depression, and even suicide, significantly affecting patients' quality of life. The principal symptoms include headache, neuralgia, and fibromyalgia without clear tissue pathology. Epidemiological estimates indicate that chronic pain prevalence ranges from below 1% to 76% worldwide (1). According to the WHO, the prevalence of chronic pain is 41% in developing countries and 37% in developed countries (2). Chronic pain frequently coexists with psychological disorders such as anxiety and depression, with comorbidity rates reported at 50% and 54%, respectively (1). This bidirectional relationship suggests that pain can exacerbate anxiety and depression, while these conditions can further intensify pain perception.

Given the high prevalence and impact of chronic pain, understanding the brain mechanisms involved in pain perception and its emotional components is crucial. The anterior cingulate cortex (ACC), a key structure in the brain's limbic system, has extensive connections with various cortical and subcortical regions and plays a central role in cognition, emotion, and motivation (3). The ACC receives afferent inputs from the medial thalamic nuclei and is involved in processing pain and emotion-related information (4). Anatomical studies have shown that ACC neurons form bidirectional connections with the amygdala, which is responsible for processing emotional and anxiety-related signals (5). This interplay makes the ACC a crucial target for investigating the neural mechanisms underlying chronic pain and its associated emotional disturbances.

Neuroinflammation, primarily mediated by microglia, astrocytes, and mast cells, is recognized as a key pathological process in chronic pain and anxiety (6, 7, 8). While both microglia and astrocytes contribute to neuroinflammation, astrocytes play a more sustained role in chronic pain maintenance, particularly in the ACC (9), Recent studies suggest that astrocyte activation exacerbates pain-related behaviors and anxiety (10, 11). Understanding how astrocyte-mediated neuroinflammation contributes to chronic pain may provide novel therapeutic targets.

The JAK/STAT pathway is a critical signaling cascade that regulates inflammation and neural plasticity (12). This pathway is implicated in various neurological disorders, including neuroinflammation, autoimmune diseases, and chronic inflammatory conditions (13, 14, 15). Several studies indicate that JAK/STAT signaling directly influences nociceptive processing and pain hypersensitivity (12). Moreover, astrocyte activation *via* the JAK2/STAT3 pathway has been specifically linked to chronic pain maintenance (16, 17, 18). Targeting this pathway may offer a promising therapeutic strategy for neuroinflammatory pain conditions.

Traditional Chinese Medicine (TCM) has been a rich source of bioactive compounds for centuries, with numerous herbal formulations demonstrating clinical efficacy and safety (19). TCM formulae typically consist of multiple medicinal herbs or minerals that act synergistically to achieve holistic therapeutic effects (20). Inspired by this principle and our previous research (21, 22, 23, 24, 25, 26), we developed a novel drug design strategy termed Combination of Traditional Chinese Medicine Molecular Chemistry (27). This approach enables the identification and optimization of bioactive compounds from herbal sources through rational chemical modifications. In a previous study (28), we designed and synthesized tanshinol borneol ester (DBZ, 1,7,7-trimethylbicyclo[2.2.1]heptan-2-yl-3-(3,4-dihydroxyphenyl)-2-hydroxy-propanoate), a key bioactive derivative inspired by the traditional herbs tanshinol and borneol. The structural formula of DBZ is in Figure 1. Our prior studies demonstrated that DBZ is a multitarget compound with diverse biological activities, including anti-inflammatory (29), pro-angiogenic (30), anti-atherosclerotic (31, 32), antioxidant (33), and cardiovascular protective effects (13). Pharmacological studies have confirmed that DBZ can cross the blood–brain barrier and reduce oxidative stress by inhibiting malondialdehyde (MDA) formation, thereby exerting neuroprotective effects (33). Additionally, DBZ has shown antineuroinflammatory properties in murine models of neuroinflammation and cerebral ischemia (29). Despite these promising findings, DBZ’s potential role in chronic pain and its associated mechanisms remains unexplored.

**Figure 1:**
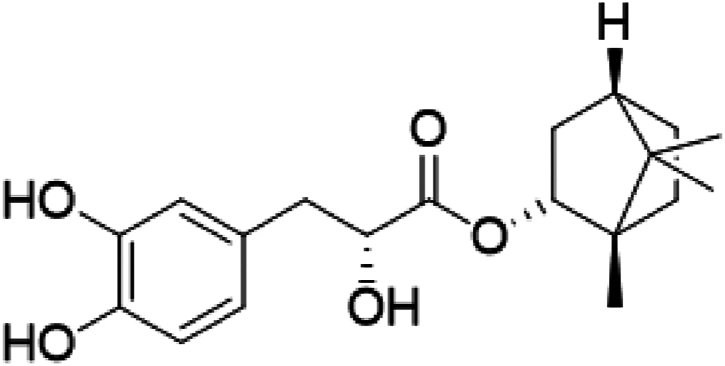
**The structural formula of DBZ.** DBZ, tanshinol borneol ester.

This study aims to address this gap by systematically evaluating the effects of DBZ in a complete Freund’s adjuvant (CFA)–induced chronic pain model. Specifically, we assess DBZ’s impact on pain-related behaviors and anxiety-like symptoms, its modulation of astrocyte-mediated neuroinflammation, and its regulation of the JAK2-STAT3 signaling pathway. Furthermore, this study integrates network pharmacology, molecular docking, and *in vivo*/*in vitro* experimental validation, providing a comprehensive mechanistic exploration of DBZ’s effects. By elucidating DBZ’s role in chronic pain management, our findings may contribute to the development of novel therapeutic strategies targeting neuroinflammatory pain conditions.

## Results

### DBZ alleviates chronic inflammatory pain induced by CFA injection

**Figure 2:**
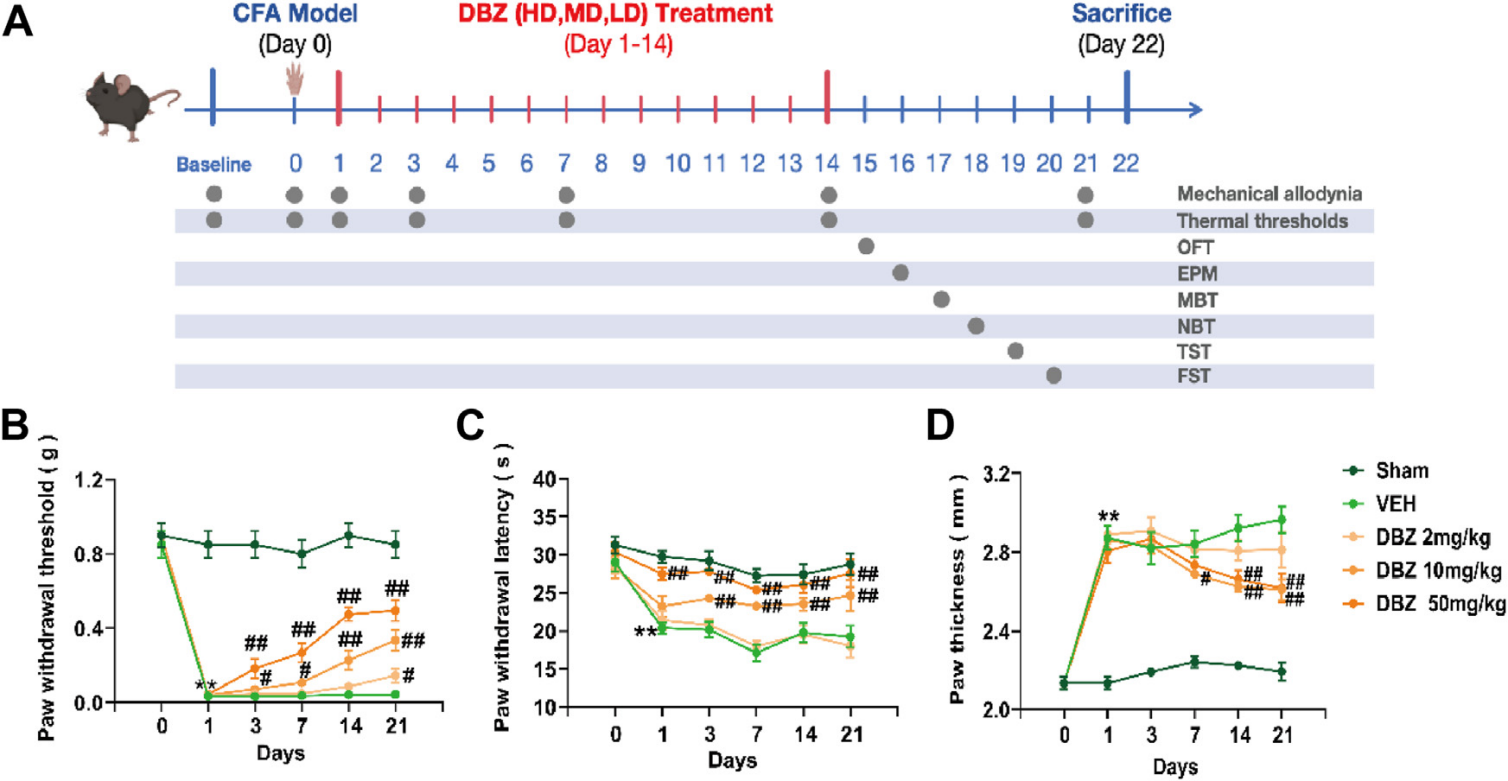
**DBZ relieved inflammatory pain in CFA mice model.***A*, experimental design is illustrated schematically. Mice received a subcutaneous injection of 10 μl of CFA (50% in saline) into the left paw to induce chronic inflammatory pain, then DBZ (2, 10, 50 mg/kg) administered intraperitoneally once daily for 14 consecutive days. *B*–*D*, Von Frey filaments to evaluate (*B*) paw withdrawal threshold (PWT), (*C*) hot plate to evaluate paw withdrawal latency, and (*D*) paw thickness measured with vernier calipers before (Day 0) and after the CFA injection (Days 1, 3, 7, 14, and 21). Open field test (OFT) on Day 15, the elevated plus maze (EPM) on Day 16, the marble burying test (MBT) on Day 17, the nest building test (NBT) on Day 18, the tail suspension test (TST) on Day 19, and the forced swimming test (FST) on Day 20, respectively were carried out. Date are presented as mean ± SEM, n = 8; ∗*p* < 0.05, ∗∗*p* < 0.01 compared to Sham, ^#^*p* < 0.05, ^##^*p* < 0.01 compared to vehicle. Statistical analyses used one-way ANOVA with *post hoc* tests. CFA, complete Freund’s adjuvant; DBZ, tanshinol borneol ester.

Intraplantar injection of CFA significantly decreased paw withdrawal latency, indicating the onset of mechanical allodynia and thermal hyperalgesia. Notably, DBZ treatment at 10 mg/kg and 50 mg/kg from Day 3 significantly increased paw withdrawal latency in response to mechanical stimuli, demonstrating its efficacy in alleviating mechanical allodynia. A lower dose of DBZ (2 mg/kg) showed effectiveness only from Day 14 (Fig. 2*B*). For thermal hyperalgesia, a 50 mg/kg dose of DBZ significantly increased paw withdrawal latency from Day 1, while a 10 mg/kg dose was effective from Day 3 (Fig. 2*C*). Moreover, DBZ markedly reduced CFA-induced toe swelling, an indicator of inflammatory edema (Fig. 2*D*). These results suggest that DBZ effectively mitigates both pain sensitivity and inflammation associated with chronic inflammatory pain.

### DBZ alleviates anxiety-like behaviors induced by CFA injection

**Figure 3:**
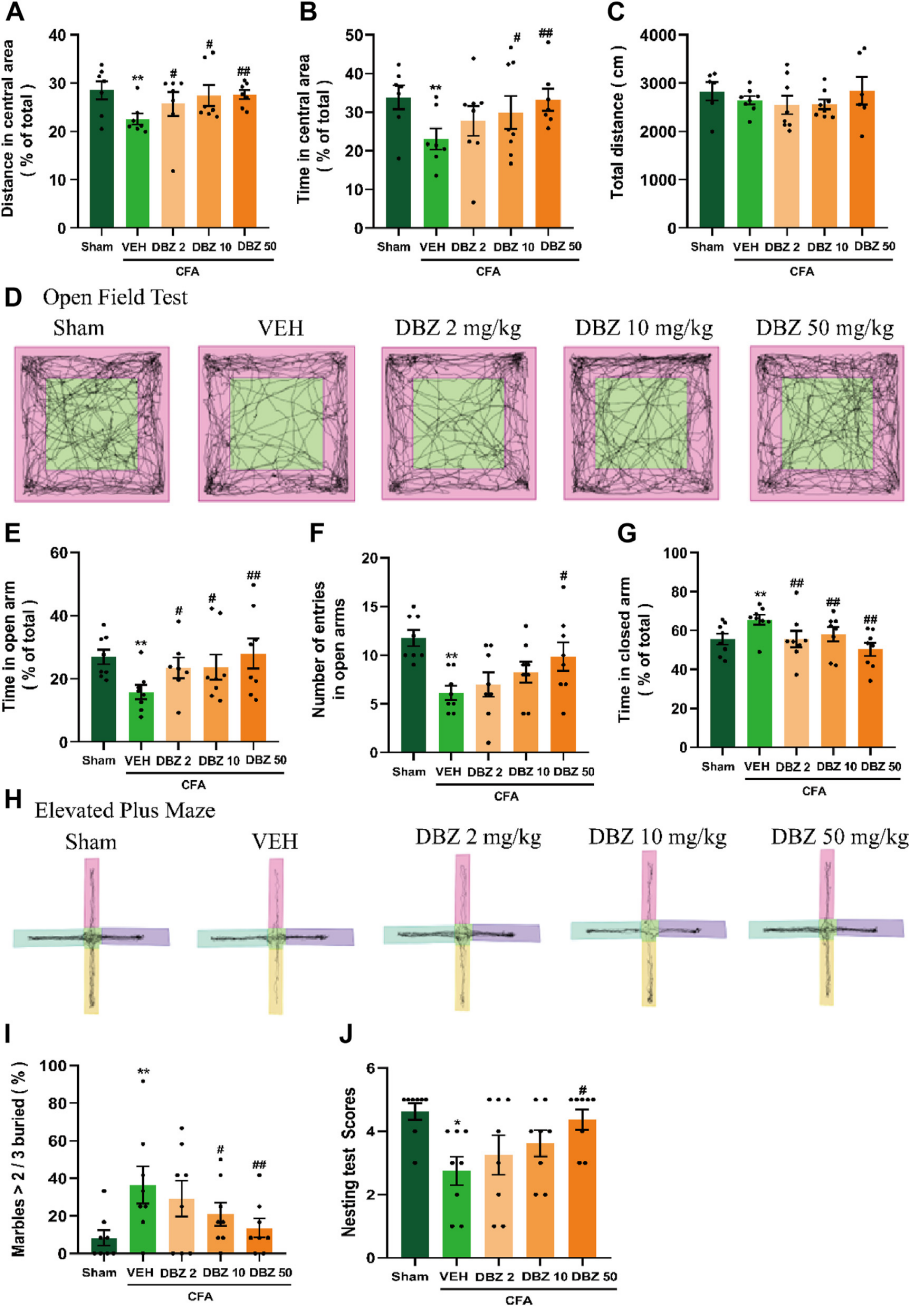
**DBZ relieved anxiety-like behaviors induced by CFA mice model.***A–C*, DBZ administration effectively reversed the reduction in the distance traveled (*A*) and the time spent (*B*) in the central area in the OFT after CFA, while the total distance traveled showed no significant different in each group (*C*). *D*, representative traces of locomotor activity in the OFT. *E*–*G*, DBZ treatment obviously increased the percentage of time spent in (*E*) and the number of entries into (*F*) the open arm and decreased the percentage of time spent in (*G*) the closed arms in the EPM test. *H*, representative traces of locomotor activity in the EPM. *I* and *J*, representative bury marbles teat (*I*) and representative nest building test (*J*). Date are presented as mean ± SEM, n = 8 mice per group; ∗*p* < 0.05, ∗∗*p* < 0.01 *versus* control group; ^#^*p* < 0.05, ^##^*p* < 0.01 *versus* model group. Statistical analyses used one-way ANOVA with *post hoc* tests. CFA, complete Freund’s adjuvant; DBZ, tanshinol borneol ester; OFT, open field test; EPM, elevated plus maze.

The anxiolytic effects of DBZ were evaluated using the open field test (OFT), elevated plus maze (EPM), burying marbles test (BMT), and nest building test (NBT). Mice injected with CFA exhibited anxiety-like behaviors, as evidenced by reduced time spent and distance traveled in the central area of the OFT compared to controls. DBZ treatment significantly and dose-dependently ameliorated these anxiety-like behaviors (Fig. 3, *A*–*D*). Importantly, total distance traveled did not differ significantly among the groups (Fig. 3*C*), indicating that DBZ did not impair locomotor activity. In the EPM test, CFA-injected mice showed reduced time and entries into the open arms, coupled with increased time spent in the closed arms, consistent with heightened anxiety. DBZ treatment effectively reversed these anxiety-associated behaviors (Fig. 3, *E*–*H*). Furthermore, in the BMT and NBT tests, CFA-injected mice buried more marbles and had lower nesting scores, both indicative of anxiety-like behaviors. DBZ treatment dose-dependently reversed these effects as well (Fig. 3, *I* and *J*), reinforcing its anxiolytic potential in CFA-induced anxiety models. Depression-like behavior was not induced by CFA (Results not shown). Notably, to determine whether DBZ *per se* affects baseline behavioral parameters in the absence of CFA-induced inflammation, we administered DBZ (50 mg/kg) to normal mice (without CFA injection) and assessed their mechanical and thermal pain thresholds, along with various anxiety-/depression-like behavioral tests. The results showed that, compared with the control group, DBZ did not significantly alter the pain thresholds, locomotor activity, or anxiety-/depression-like behavior scores in normal mice (*p* > 0.05). These findings suggest that DBZ itself does not affect baseline behavioral and emotional parameters under normal conditions.

### Network pharmacology-based analysis

**Figure 4:**
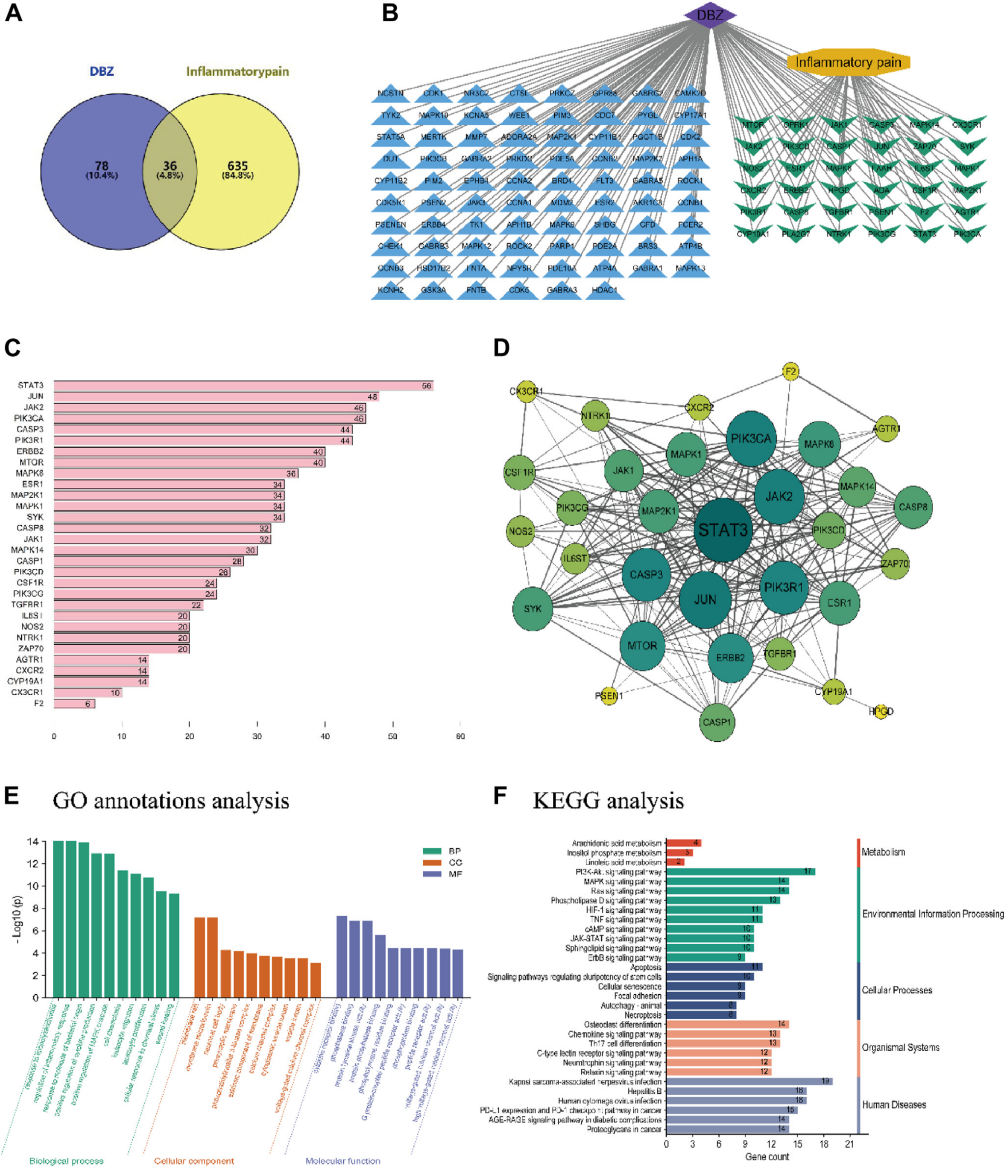
**Network pharmacology of DBZ and inflammatory pain.***A*–*F*, Venn diagram of overlap genes in potential targets of DBZ and inflammation pain (*A*), DBZ and inflammation pain common targets (*B*), degree of interaction of common targets (*C*), PPI network of DBZ and inflammation pain common targets (*D*), GO enrichment analysis for potential targets (*E*), KEGG enrichment analysis for potential targets (*F*). DBZ, tanshinol borneol ester; GO, gene ontology; PPI, protein-protein interactions.

Following standardization *via* Uniprot, 36 overlapping targets between DBZ and inflammation-induced pain were obtained (Fig. 4*A*).

The protein-protein interactions network consists of 35 nodes and 225 edges, with an average node degree of 12.9 and a clustering coefficient of 0.664, indicating a highly interconnected system (Fig. 4*B*). Figure 4*C* highlights the top 30 intersection targets ranked by degree, with STAT3, JAK2, JUN, PIK3CA, and CASP3 ranking as the top five targets with the highest degree values. These targets are considered key hub genes due to their high connectivity and potential regulatory roles in inflammation and pain signaling pathways (Fig. 4*D*), suggesting their importance in the molecular mechanisms of DBZ in treating inflammatory pain.

The top three CCs include membrane raft, neuronal cell body, and phosphatidylinositol 3-kinase complex (Fig. 4*E*). For MF, the top three terms include cytokine receptor binding, protein tyrosine kinase activity, and phosphotyrosine residue binding. In terms of BP, notable processes include regulation of inflammatory response and positive regulation of cytokine production.

The top five pathways include PI3K-AKT, Phospholipase D, TNF, MAPK, and JAK-STAT signaling pathways (Fig. 4*F*), all of which are critical to inflammatory processes and immune responses. These findings further support the hypothesis that DBZ modulates inflammation-related pathways, with particular emphasis on the JAK-STAT signaling pathway.

### DBZ suppresses inflammation and oxidative stress in serum and ACC

**Figure 5:**
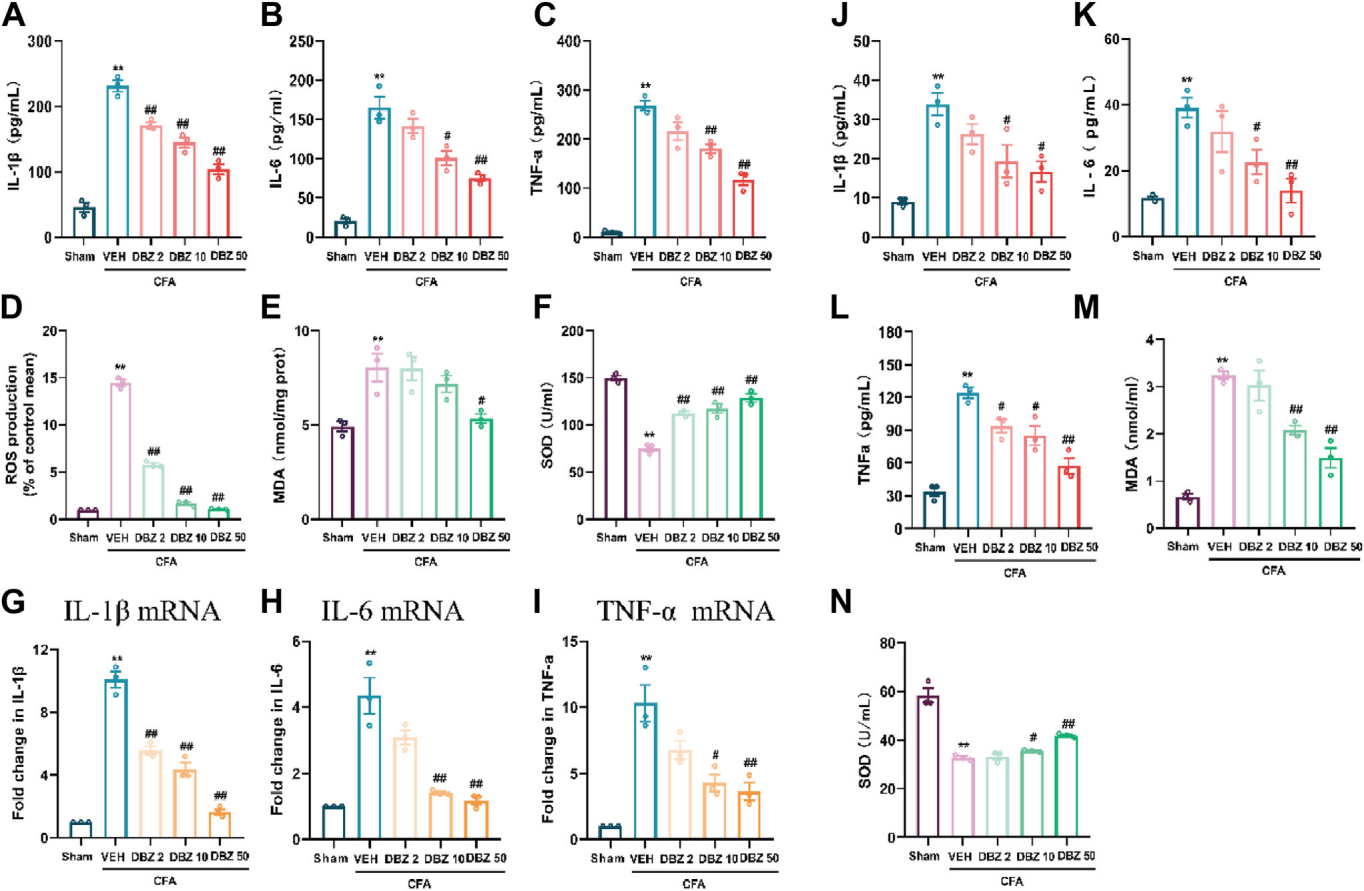
**DBZ suppresses inflammation and oxidative stress in the ACC and serum.***A*–*I*, DBZ suppresses IL-1β (*A*), IL-6 (*B*), and TNF-a (*C*) in the ACC, suppresses ROS (*D*), MDA (*E*), and enhance SOD (*F*) in the ACC, suppresses IL-1β mRNA (*G*), IL-6 mRNA (*H*), and TNF-a mRNA (*I*) of CFA-injected mice*. J–N*, DBZ suppresses IL-1β (*J*), IL-6 (*K*), and TNF-a (*L*) in the serum, suppresses MDA (*M*), and enhances SOD (*N*). Date are presented as mean ± SEM, n = 3, ∗*p* < 0.05 and ∗∗*p* < 0.01 *versus* Sham, ^#^*p* < 0.05 and ^##^*p* < 0.01 *versus* VEH. Statistical analyses used one-way ANOVA with *post hoc* tests. ACC, anterior cingulate cortex; CFA, complete Freund’s adjuvant; DBZ, tanshinol borneol ester; MDA, malondialdehyde; ROS, reactive oxygen species; SOD, superoxide dismutase.

Inflammation and oxidative stress are critical factors contributing to chronic pain and anxiety. To evaluate the effect of DBZ on these factors, we measured the levels of pro-inflammatory cytokines (IL-1β, IL-6, and TNF-α) in both serum and the ACC using enzyme-linked immunosorbent assay (ELISA). In the ACC, CFA injection significantly increased the levels of IL-1β, IL-6, and TNF-α (Fig. 5, *A*–*C*), IL-1β mRNA, IL-6 mRNA, TNF-a mRNA (Fig. 5, *G*–*I*), reactive oxygen species (ROS), MDA (Fig. 4, *D* and *E*), and reduced superoxide dismutase (SOD) (Fig. 4*F*), compared to the control group. DBZ treatment reversed these cytokine levels in a dose-dependent manner, with the 50 mg/kg dose showing the most significant reverse the level in these factors. Similarly, in the serum, CFA injection elevated IL-1β, IL-6, and TNF-α (Fig. 5, *J*–*L*), MDA (Fig. 5*M*), and reduced SOD (Fig. 5*N*). DBZ treatment at 10 mg/kg and 50 mg/kg significantly reversed the levels of these factors. These results suggest that DBZ exerts a potent anti-inflammatory and antioxidant effect, potentially contributing to its ability to alleviate chronic pain and anxiety-like behaviors in CFA-injected mice.

### Effects of DBZ on glutamate receptor levels and AK2/STAT3 signaling pathway in ACC

**Figure 6:**
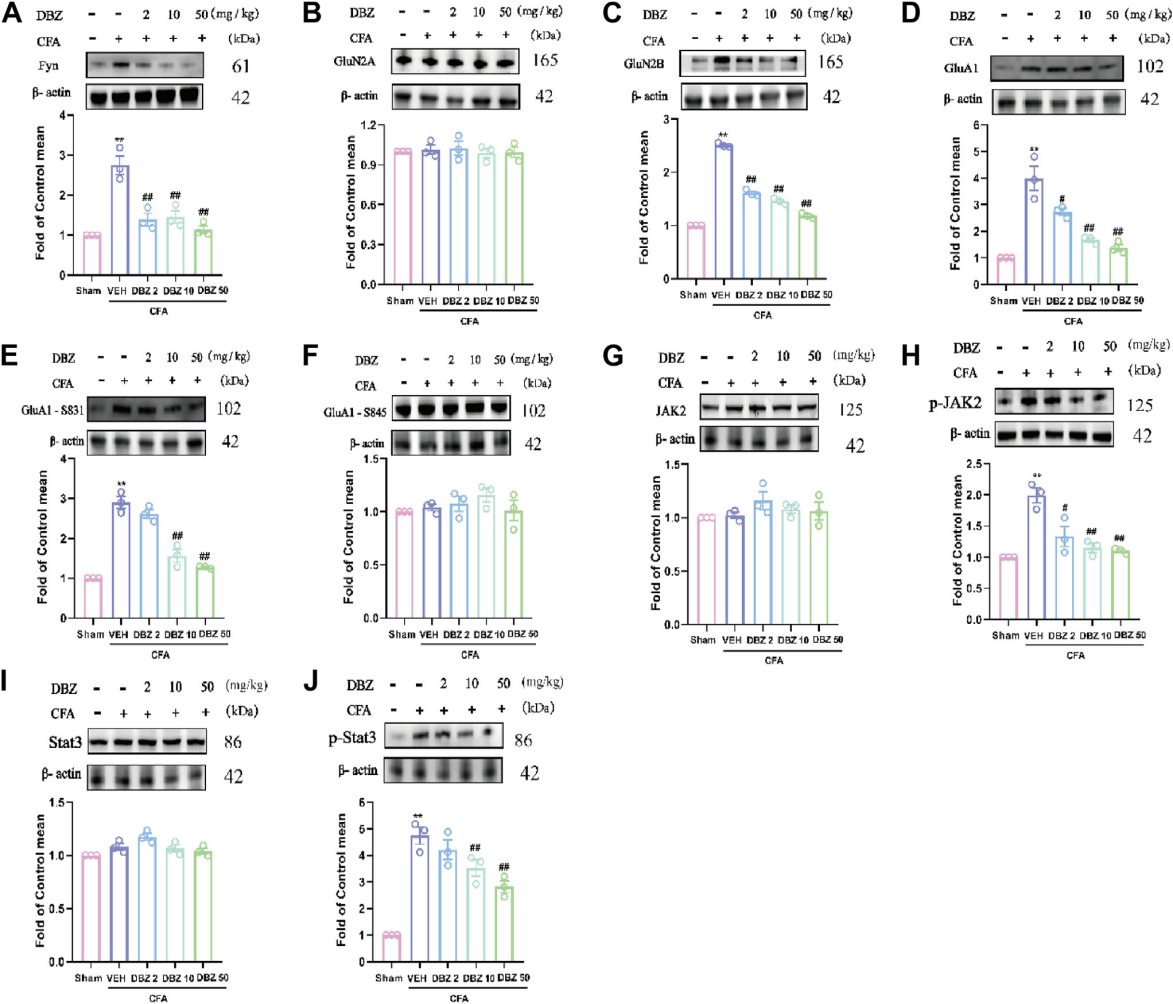
**DBZ improved changes in Fyn, glutamate receptor and JAK2/STAT3 expression in the ACC of CFA-injected mice.***A*–*F*, DBZ treatment reversed the increased expression of Fyn (*A*), GluN2B (*C*), GluA1 (*D*), GluA1-S831(*E*) but had no apparent effect on GluN2A (*B*) and GluA1-S45 (*F*) normalized to β-actin. DBZ also improved changes in JAK2 and STAT3 receptor expression in the ACC of CFA-injected mice. *G*–*J*, DBZ treatment reversed the increased expression of p-JAK2 (*H*), p-STAT3 (*J*) but had no apparent effect on JAK2 (*G*) and STAT3 (*I*) normalized to β-actin. Date are presented as mean ± SEM, n=3 mice, ∗*p* < 0.05 and ∗∗*p* < 0.01 *versus* Sham, ^#^*p* < 0.05 and ^##^*p* < 0.01 *versus* VEH. Statistical analyses used one-way ANOVA with post hoc tests. ACC, anterior cingulate cortex; CFA, complete Freund’s adjuvant; DBZ, tanshinol borneol ester.

The effects of DBZ on NMDA and AMPA receptor levels were examined in the ACC, as depicted in Figure 6. Regarding NMDA receptor subtypes, GluN2B expression was markedly upregulated following CFA induced (Fig. 6*C*), while GluN2A levels were unaffected (Fig. 6*B*). CFA injection in the ACC led to a significant increase in GluA1 expression (Fig. 6*D*) and its phosphorylation at Ser831 (p-GluA1-Ser831) (Fig. 6*E*), indicating an enhanced AMPA receptor activity. However, the phosphorylation of GluA1 at Ser845 (p-GluA1-Ser845) remained largely unchanged by CFA treatments (Fig. 6*F*). This pattern aligns with the observed increase in Fyn expression (Fig. 6*A*), a kinase known to modulate GluN2B phosphorylation and activity. DBZ treatment demonstrated a dose-dependent reversal of these alterations in ACC. Specifically, DBZ significantly reduced the elevated levels of Fyn, GluN2B, GluA1, and p-GluA1-Ser831 in the ACC at higher doses at 10 and 50 mg/kg. Notably, DBZ did not affect GluN2A expression or the phosphorylation of GluA1 at Ser845 under these conditions. These findings suggest that DBZ mitigates the hyperexcitatory transmission induced by CFA in the ACC, predominantly through its regulatory effects on the GluA1 and GluN2B subunits, as well as Fyn kinase activity.

To investigate whether the JAK2/STAT3 pathway is involved in DBZ-mediated inactivation of chronic inflammation pain and inhibition of inflammation and oxidative stress, we examined the expression of proteins associated with the JAK2/STAT3 pathway *via* western blot analysis. The results showed a significant increase in the phosphorylation of JAK2 and STAT3 (Fig. 6, *H* and *J*) after CFA injection in the ACC. However, DBZ treatment blocked the activation of the JAK2/STAT3 pathway, suggesting that the DBZ-mediated effects on chronic inflammation pain may be associated with the JAK2/STAT3 pathway.

### DBZ suppresses astrocyte activation in the ACC of CFA-injected mice

**Figure 7:**
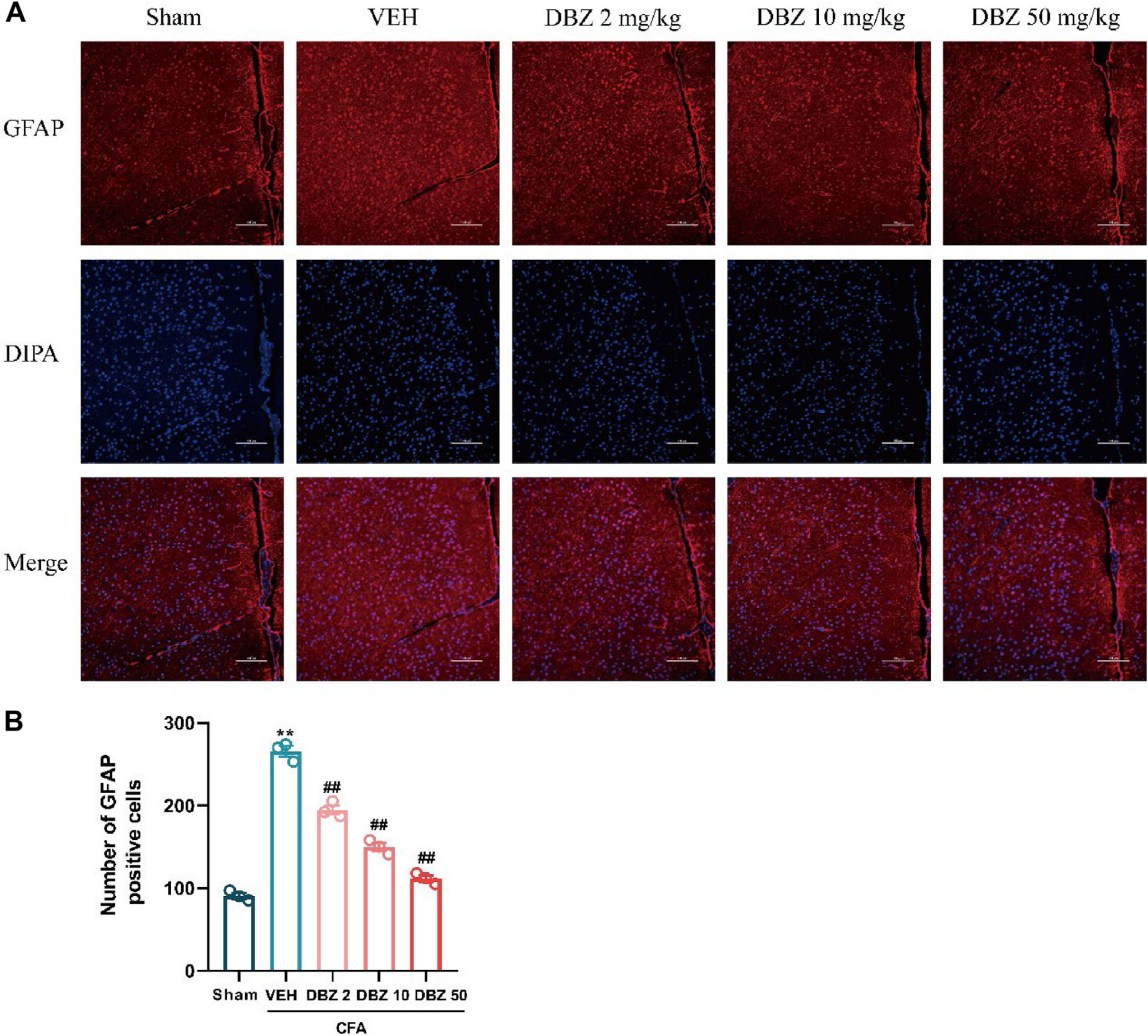
**DBZ reduced astrocytes activation in the ACC of CFA-injected mice.***A*, slices were immunostained using the astrocytes marker GFAP antibody (*red*), and nuclei stained with DIPA (*blue*). Scale bar = 100 μm. *B*, DBZ inhibited the activation of astrocytes in the ACC after CFA injection and had a dose-dependent effect. Data are presented as mean ± SEM, n = 3 mice, ∗*p* < 0.05 and ∗∗*p* < 0.01 *versus* Sham, ^#^*p* < 0.05 and ^##^*p* < 0.01 *versus* VEH. Statistical analyses used one-way ANOVA with post hoc tests. ACC, anterior cingulate cortex; CFA, complete Freund’s adjuvant; DBZ, tanshinol borneol ester; GFAP, glial fibrillary acidic protein.

Astrocytes are key mediators of neuroinflammation, particularly through the expression of glial fibrillary acidic protein (GFAP). Immunofluorescence staining was used to assess astrocyte activation in the ACC of CFA-injected mice. As shown in Fig. 7*A*, there was a significant increase in GFAP-positive cells in the ACC of the model group, indicating astrocyte activation in response to inflammation. DBZ treatment markedly reduced the number of GFAP-positive astrocytes in a dose-dependent manner. Specifically, the group treated with 50 mg/kg DBZ showed the most substantial reduction in astrocyte activation compared to the model group, followed by the 10 mg/kg group, and the 2 mg/kg group showed a moderate reduction (Fig. 7*A*). The quantitative analysis of GFAP-positive cells is shown in Fig. 7*B*, further confirming that DBZ effectively suppresses astrocyte activation in the ACC. These results suggest that DBZ not only alleviates peripheral inflammation but also has a significant impact on neuroinflammation, potentially contributing to the relief of chronic pain and anxiety symptoms in CFA-injected mice.

### DBZ suppresses inflammation and oxidative stress in primary astrocytes

**Figure 8:**
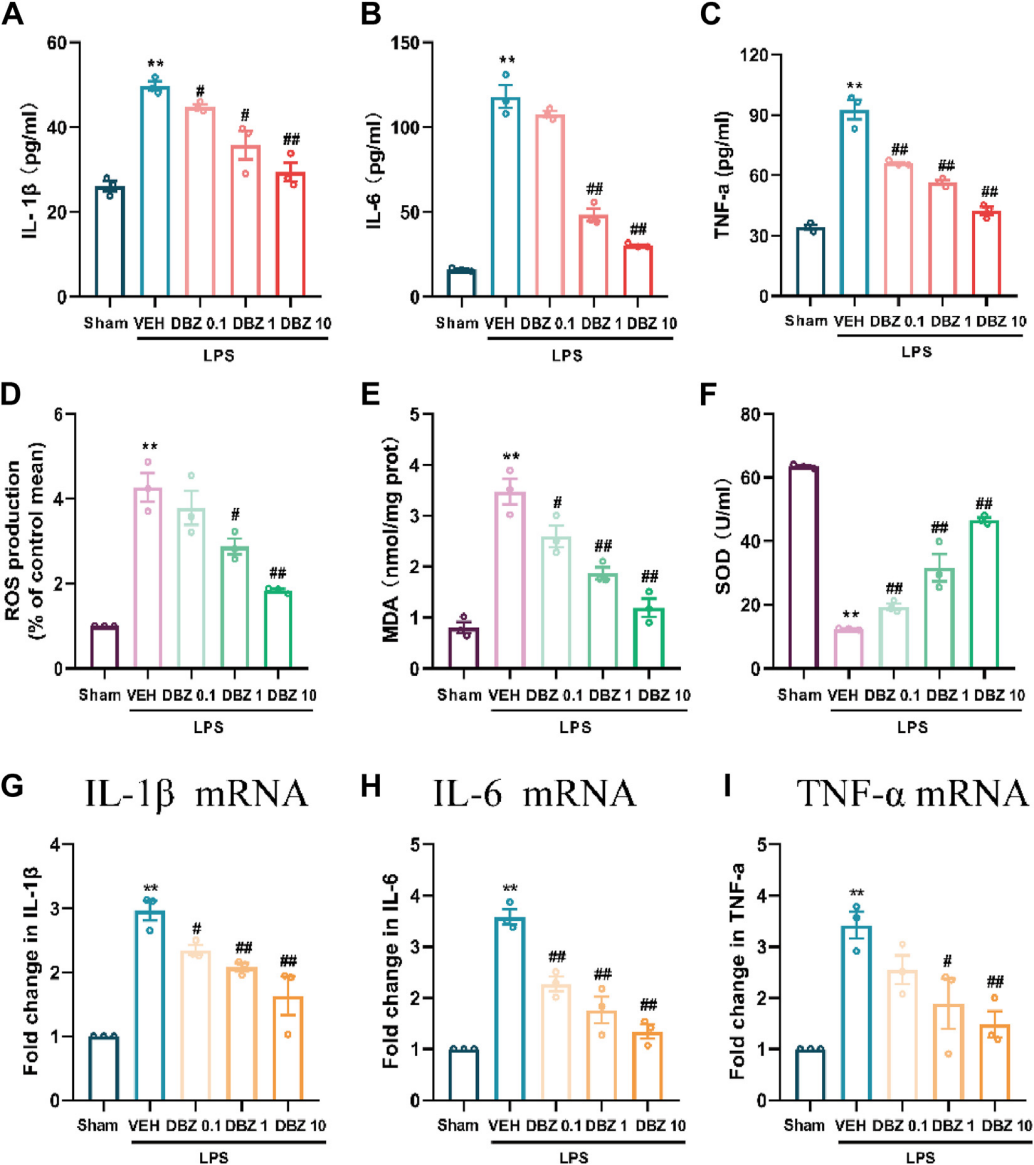
**DBZ suppresses inflammation and oxidative stress in the primary astrocytes.***A*–*I*, DBZ suppresses IL-1β (*A*), IL-6 (*B*), and TNF-a (*C*) and ROS (*D*), MDA (*E*), and enhance SOD (*F*) in the primary astrocytes. DBZ also suppresses IL-1β mRNA (*G*), IL-6 mRNA (*H*) and TNF-α mRNA (*I*). Data are presented as mean ± SEM, n = 3, ∗*p* < 0.05 and ∗∗*p* < 0.01 *versus* Sham, ^#^*p* < 0.05 and ^##^*p* < 0.01 *versus* VEH. Statistical analyses used one-way ANOVA with *post hoc* tests. Loading controls were selected based on experimental optimization: GAPDH or beta-actin was used as indicated. DBZ, tanshinol borneol ester; MDA, malondialdehyde; ROS, reactive oxygen species; SOD, superoxide dismutase.

In this study, the anti-inflammatory effects of DBZ on lipopolysaccharide (LPS)-stimulated primary astrocytes were evaluated. As shown in Figure 8, DBZ treatment significantly inhibited the production of key pro-inflammatory cytokines, including IL-1β, IL-6, and TNF-α (Fig. 8, *A*–*C*), and IL-1β mRNA, IL-6mRNA, and TNF-α mRNA (Fig. 8, *G*–*I*) in a dose-dependent manner. In addition to reducing cytokine levels, DBZ was found to attenuate LPS-induced oxidative stress, as indicated by reductions in ROS, MDA, and increased SOD levels (Fig. 8, *D*–*F*). Notably, DBZ exerted these protective effects without any observable cytotoxicity at the 10 μM highest concentration tested (data were not shown). These results suggest that DBZ exerts potent anti-inflammatory and antioxidant effects on astrocytes, further supporting its role in alleviating neuroinflammation and oxidative stress, both of which are critical in the pathogenesis of chronic pain and anxiety-like behaviors.

### Effects of DBZ on glutamate receptor levels and JAK2/STAT3 pathway in the primary astrocytes

**Figure 9:**
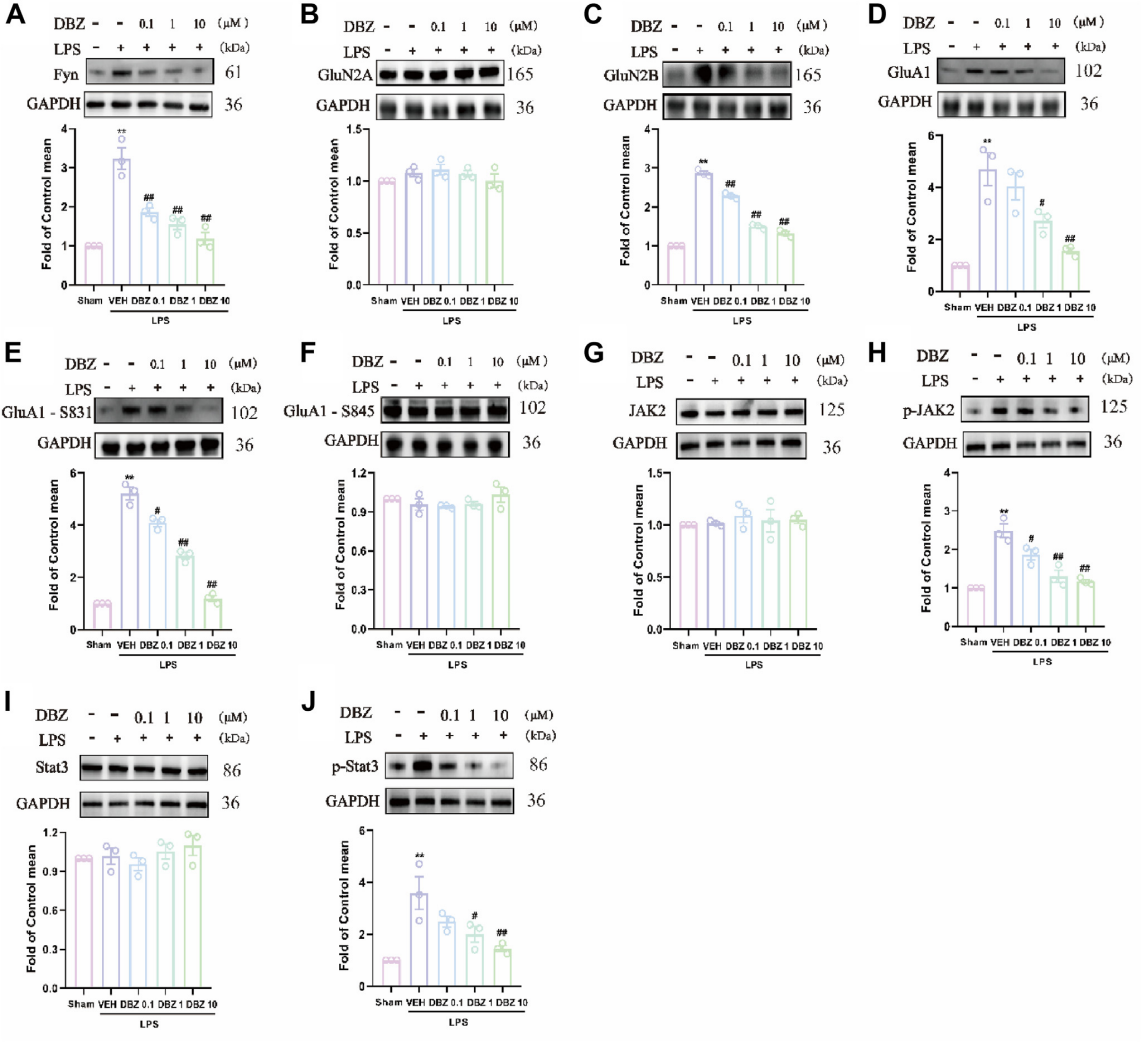
**DBZ improved changes in Fyn and glutamate receptor and JAK2/STAT3 expression in the primary astrocytes of LPS induced.***A*–*F*, DBZ treatment reversed the increased the increased expression of Fyn (*A*), GluN2B (*C*), GluA1 (*D*), GluA1-S831 (*E*) but had no apparent effect on GluN2A (*B*) and GluA1-S45 (*F*) normalized to GAPDH. DBZ also improved changes in JAK2 and STAT3 receptor expression in the primary astrocytes of LPS induced. *G*–*J*, DBZ treatment reversed the increased the increased expression of p-JAK2 (*H*), p-STAT3 (*J*) but had no apparent effect on JAK2 (*G*) and STAT3 (*I*) normalized to β-actin. Data are presented as mean ± SEM, n = 3, ∗*p* < 0.05 and ∗∗*p* < 0.01 *versus* Sham, ^#^*p* < 0.05 and ^##^*p* < 0.01 *versus* VEH. Statistical analyses used one-way ANOVA with post hoc tests. DBZ, tanshinol borneol ester; LPS, lipopolysaccharide.

As depicted in Figure 9, GluN2B expression was markedly upregulated following LPS stimulation (Fig. 9*C*), while GluN2A levels were unaffected (Fig. 9*A*). LPS induction led to a significant increase in GluA1 expression (Fig. 9*D*) and its phosphorylation at Ser831 (p-GluA1-Ser831) (Fig. 9*E*), indicating an enhanced AMPA receptor activity. However, the phosphorylation of GluA1 at Ser845 (p-GluA1-Ser845) remained largely unchanged by LPS treatments (Fig. 9*F*). This pattern aligns with the observed increase in Fyn expression (Fig. 9*A*), a kinase known to modulate GluN2B phosphorylation and activity. DBZ treatment demonstrated a dose-dependent reversal of these alterations. These findings suggest that DBZ mitigates the hyperexcitatory transmission induced by LPS in primary astrocytes, predominantly through its regulatory effects on the GluA1 and GluN2B subunits, as well as Fyn kinase activity.

Same as ACC, the results showed a significant increase in the phosphorylation of JAK2 and STAT3 after LPS stimulation (Fig. 9, *H* and *J*). However, DBZ treatment blocked the activation of the JAK2/STAT3 pathway, suggesting that the DBZ-mediated effects on chronic inflammation pain may be associated with the JAK2/STAT3 pathway.

### Molecular docking simulation

**Figure 10:**
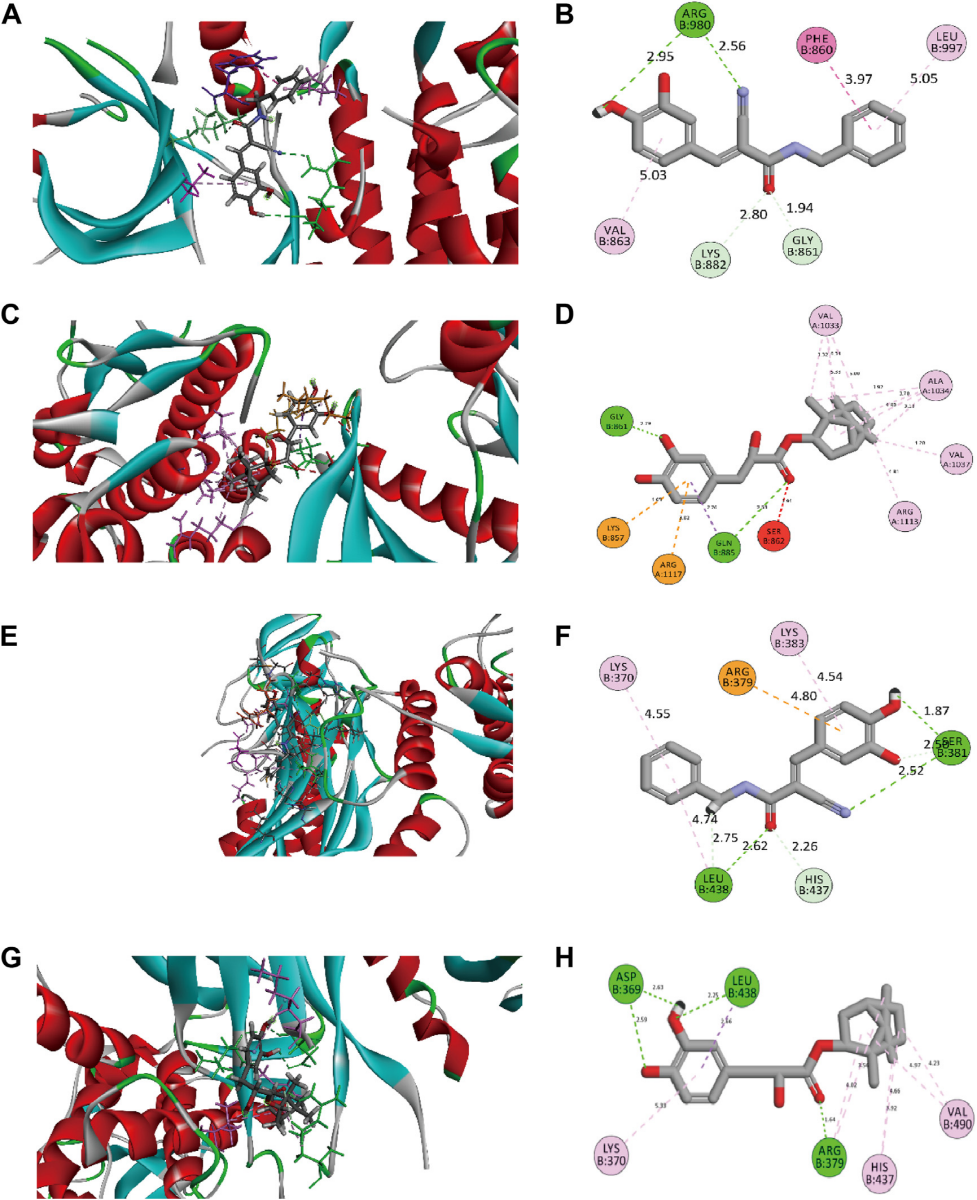
**Binding interactions of AG490 and DBZ with JAK2 and STAT3.***A*–*H*, the 3D interaction diagrams illustrate key binding sites between AG490 with JAK2 (*A* and *B*), DBZ with JAK2 (*C* and *D*) and AG490 with STAT3 (*E* and *F*), DBZ with STAT3 (*G* and *H*). DBZ interacts with JAK2 (PDB code: 4HGE) (*C*) and STAT3 (PDB code: 6TLC) (*G*). *Colored dashed lines* represent interactions between DBZ and amino acids, nucleic acids, or metals. The *green spline curves* indicate regions of hydrophobic interaction. The 3D interaction diagram shows the major binding sites between AG490 with JAK2 (*B*) and STAT3 (*F*), DBZ with JAK2 (*D*) and STAT3 (*H*). DBZ, tanshinol borneol ester.

Molecular docking simulations were conducted to evaluate the binding affinity of AG490 and DBZ to key proteins in the JAK-STAT signaling pathway. The results revealed significant binding interactions between DBZ and JAK2 (Fig. 10, *C* and *D*), as well as STAT3 (Fig. 10, *G* and H). The binding mode of DBZ was similar to that of the AG490 with JAK2 (Fig. 10, *A* and *B*) and STAT3 (Fig. 10, *E* and *F*). These findings suggest that DBZ forms stable complexes with both JAK2 and STAT3, potentially modulating the signaling pathway. This interaction likely represents a key mechanism underlying the anti-inflammatory effects of DBZ, further validating that DBZ exerts its therapeutic effects through these molecular targets.

## Discussion

Current treatments for neuroinflammatory diseases offer only partial relief, underscoring the urgent need for more effective therapeutic strategies (29). Astrocytes, the most abundant glial cells in the central nervous system (CNS), perform a wide range of homeostatic functions (34). These functions can be disrupted in neurological disorders, and the activation of specific astrocyte populations contributes to the pathology of conditions such as multiple sclerosis, Alzheimer’s disease, Parkinson’s disease, Huntington’s disease, and chronic pain (35, 36, 37, 38). This highlights the critical roles of astrocytes in CNS repair. Additionally, the interplay between oxidative stress and inflammation can create a vicious cycle that exacerbates neural damage (39).Therefore, compounds with antioxidant and anti-inflammatory properties may offer therapeutic benefits for chronic pain and neuroinflammation.

Our study demonstrates that DBZ alleviates chronic inflammation pain and anxiety behaviors induced by CFA injection. These findings are particularly significant as they highlight DBZ’s potential as a novel therapeutic agent in addressing both pain and anxiety associated with chronic inflammation. DBZ exhibited antioxidant and anti-inflammatory effects in both CFA-induced mouse models and primary astrocytes. Mechanistic studies revealed that these effects are strongly associated with the JAK2/STAT3/Fyn/NMDA signaling pathway. The involvement of the JAK2/STAT3/Fyn/NMDA signaling pathway provides insight into how DBZ modulates neuroinflammation and pain pathways, potentially offering a multitargeted approach to therapy. While molecular docking provides valuable insights into DBZ’s potential interaction with JAK2/STAT3, direct *in vitro* validation remains to be established. Future studies will employ co-immunoprecipitation and other biochemical approaches to confirm these interactions and further elucidate DBZ’s molecular mechanism.

The ACC is involved in signaling the unpleasantness of pain, and hyperactivation of the ACC has been consistently observed in functional MRI studies of individuals with neuropathic and chronic visceral pain (40). Synaptic plasticity, particularly in the ACC, has been extensively studied due to its role in pain processing. Nociceptive information from somatic and visceral organs is conveyed to the ACC through the thalamus (41), amygdala (42), and other related cortical areas. Neurons in the ACC respond to both noxious and non-noxious mechanical or thermal stimuli. Optogenetic studies have shown that activating pyramidal neurons in the ACC can lower mechanical pain thresholds in mice (43). However, in a mouse model of inflammation pain induced by CFA injection, optogenetic stimulation of ACC pyramidal neurons did not further lower the mechanical threshold, suggesting that ACC pyramidal neuron activation alone is sufficient to mediate hypersensitivity. DBZ treatment administered for 14 consecutive days following CFA injection, a period during which astrocyte activation occurs, significantly inhibited astrocyte activation in the ACC of CFA-injected mice and reduced inflammatory cytokine release. These results suggest that DBZ may alleviate chronic inflammatory pain through its inhibitory effect on astrocytes.

Glutamate, the major excitatory neurotransmitter in the CNS, activates glutamate receptors such as AMPARs and NMDARs (44). These receptors are crucial for developing pain hypersensitivity. Under normal conditions, NMDA receptors are inactive, but injury-induced neurotransmitter release can activate these receptors by sufficiently depolarizing postsynaptic neurons (45). This activation increases calcium influx, strengthening synaptic connections between nociceptors and dorsal horn pain transmission neurons, thereby exacerbating responses to noxious stimuli (45). Studies have shown that excitatory glutamate receptors are upregulated in various mouse models (46, 47). Chronic pain can be reversed by reducing AMPARs and NMDARs in the ACC glutamatergic system (48). In this study, we observed elevated levels of GluA1-Ser831, GluN2B, and GluA1 in the ACC of CFA-injected mice and LPS-treated primary astrocytes, which were reversed by DBZ treatment. Further studies are needed to explore the direct effects of DBZ on synaptic plasticity.

In the CNS, glial cells, including astrocytes, microglia, and oligodendrocytes, constitute more than half of the total cell population, with astrocytes making up approximately 20 to 40% (49). Astrocytes not only provide structural and nutritional support for neurons but also play crucial roles in many neural processes (50). While astrocytes are generally in a resting state, they become reactive in response to tissue injury or disease, contributing to neurological disorders (51). One distinguishing feature of astrocytes is their ability to communicate directly through gap–junction protein complexes, allowing the exchange of ions and small cytosolic components (36). Injury to peripheral nerves triggers the release of TNF-α from activated microglia, stimulating astrocytes (52). Inhibition of TNF-α signaling through neutralizing antibodies or inhibitors can alleviate chronic pain (53). Similar to TNF-α, IL-1β is expressed in both microglia and astrocytes and is upregulated in astrocytes in various chronic pain conditions, including inflammatory pain (54), neuropathic pain (55), and bone cancer pain (56). Optogenetic activation of astrocytes also increases IL-1β and TNF-α secretion (57). In human studies, IL-1β and TNF-α levels are elevated in the spinal astrocytes of patients with HIV-associated chronic pain (58). Reactive astrocytes and their specific signaling pathways are differentially modulated across various CNS disorders. STAT3 signaling in reactive astrocytes has been highlighted in multiple studies (59, 60). IL-6 signaling through STAT3 regulates astrocytes linked to neurotoxicity during CNS inflammation. In traumatic injury contexts, reactive astrocytes form borders around CNS lesions, restricting inflammation (60, 61). STAT3 inactivation in astrocytes impairs locomotor recovery and increases pro-inflammatory gene transcription in spinal cord injury models, emphasizing the role of STAT3-controlled astrocyte products in CNS repair (38). Thus, STAT3 in astrocytes regulates CNS inflammation and neurodegeneration in a context-specific manner. We performed *in vitro* tests on primary astrocytes to determine whether DBZ reduces neuronal excitability through inhibition of astrocyte activation. Our results indicated that DBZ treatment significantly reduced levels of excitatory synaptic proteins in LPS-stimulated primary astrocytes. Moreover, the expression of these excitatory proteins was closely related to inflammatory cytokines TNF-α and IL-1β. These findings align with previous studies, indicating that DBZ’s analgesic effect is associated with the inhibition of astrocyte activation.

One limitation of our study is the absence of a standard anti-inflammatory or JAK2 inhibitor as a positive control. Although AG490 is commonly used to validate the JAK2/STAT3 pathway, it was not commercially available at the time of our experiments. Despite this, our data strongly suggest that DBZ mediates its anti-inflammatory and analgesic effects *via* modulation of the JAK2/STAT3 pathway. In future studies, we plan to use alternative, readily available JAK2 inhibitors (*e.g.*, ruxolitinib or baricitinib) to further validate DBZ’s molecular targets and efficacy.

In conclusion, our data reveal for the first time that DBZ relieves inflammation-induced pain and anxiety in mice and confirm that DBZ’s analgesic effect is attributed to the inhibition of astrocyte activation, blockade of pro-inflammatory cytokine release, antioxidant properties, and regulation of excitatory synaptic proteins. The involvement of the JAK2/STAT3/Fyn/NMDA signaling pathway in these effects highlights DBZ as a potential candidate for treating neurological disorders associated with neuroinflammation. These findings not only advance our understanding of DBZ’s therapeutic potential but also underscore its promise as a candidate for future clinical development in treating neuroinflammatory disorders.

## Experimental procedures

### Materials

DBZ (United States Patent No. 8017786) was synthesized in our laboratory using the method described by Bai *et al.* (28).

### Animals

Adult male C57BL/6 mice, aged 4 to 5 weeks, were purchased from the Experimental Animal Center of the Fourth Military Medical University. Upon arrival, the animals were acclimated for 1 week before the start of the experiments. They were housed under specific pathogen-free conditions in polycarbonate cages with standard sawdust bedding. The environmental conditions were controlled with a temperature of 22 to 24 °C, humidity at 55 ± 10%, and a 12-h light/dark cycle (lights on from 07:00–19:00). Mice had ad libitum access to standard laboratory chow and filtered water. Caging equipment was sterilized, food was irradiated, and water was filtered to ensure cleanliness.

All animal studies were conducted in accordance with the ARRIVE guidelines. The experiments were approved by the Ethics Committee on Animal Research and Welfare of Northwest University. Animal care and experimental procedures adhered to the regulations established by the State Committee of Science and Technology of the People’s Republic of China (November 14, 1988) and the National Institutes of Health Guide for the Care and Use of Laboratory Animals (NIH Publications No. 8023, revised 1978).

### Experimental designs and DBZ treatment

Mice were randomly divided into five groups: vehicle control group, CFA group, low-dose DBZ-treated group, middle-dose DBZ-treated group, and high-dose DBZ-treated group respectively. Mice in the CFA group and DBZ-treated groups received a subcutaneous injection of 10 μl of CFA (50% in saline) into the left paw to induce chronic inflammatory pain. The control group was injected with an equal volume of 0.9% saline into the same location. DBZ was dissolved in a 0.2% (w/v) poloxamer 188 solution and administered intraperitoneally at doses of 2.0, 10.0, and 50.0 mg/kg to the DBZ-treated groups once daily for 14 consecutive days, starting after the CFA injection. Control animals received an equivalent volume of the vehicle *via* the same route.

### Mechanical allodynia

To assess mechanical allodynia, mice were placed in individual plastic boxes for 30 min to acclimate. Mechanical allodynia was evaluated using von Frey filaments (ranging from 0.008 to 2 g) with an up-down paradigm before (Day 0) and at multiple time points after CFA injection (Days 1, 3, 7, 14, and 21). The filaments were applied to the plantar surface of the hind paws until they bent, with each filament being tested five times at 10-s intervals. Testing of the injected hind paws was performed with intervals longer than 5 min. Positive responses were characterized by prolonged hind paw withdrawal followed by licking or scratching. The force of the next filament used was selected based on the previous results.

### Thermal thresholds

Mice were placed in a quiet environment with the room temperature controlled at 24 °C ± 2 deg and allowed to acclimate for at least 30 min. Prior to testing, mice were placed on a hot plate surface under an inverted transparent plastic cage, with the temperature set at 50 °C ± 0.1 deg. Quick hind paw movements, licking, swinging, and lifting upon touching the hot plate were considered withdrawal responses. Each paw was tested with intervals of more than 5 min between measurements. Three trials were conducted for each hind paw, and the average of these trials was used as the mean thermal paw withdrawal latency. A 30-s cutoff was used to prevent tissue damage. Thermal thresholds were measured before (Day 0) and after the CFA injection (Days 1, 3, 7, 14, and 21).

### Behavioral tests

All behavioral tests were conducted in behavioral testing rooms between 9:00 and 16:00 during the light phase of the light/dark cycle. Mice were subjected to the OFT, EPM, MBT, the NBT, the tail suspension test (TST), and the forced swimming test (FST), as described in previous reports. Each mouse was acclimated to the testing room 2 h before the tests. Mice were administered once 30 min before the behavioral test. The OFT was always performed before the EPM, but tests were conducted on the same day.

#### OFT

The open field apparatus (JLbehv-LAM-4, Shanghai Jiliang Software) was a square arena (30 × 30 × 30 cm^3^) with clear Plexiglas walls and floor and placed inside an isolation chamber with dim illumination and a fan. Each mouse was placed in the center of the box and allowed to explore freely for 10 min. Exploratory behaviors were recorded using a camera fixed above the chamber. The total distance traveled and the time spent in the central area were analyzed using a video-tracking system (Med Associates).

#### EPM

The EPM apparatus (DigBehv-EPMG, Shanghai Jiliang Software) consisted of two open arms (25 × 8 × 0.5 cm^3^) and two closed arms (25 × 8 × 12 cm^3^) extending from a common central zone (8 × 8 cm^2^). Mice were handled gently twice to reduce nervousness. For each test, an individual mouse was placed in the central zone facing an open arm and allowed to explore freely for 6 min while being videotaped. The time spent in the open and closed arms was analyzed using a video-tracking system (Med Associates).

#### MBT

The MBT was conducted as described in previous reports (62). A cage (17.5 × 10 × 5.5 cm^3^) was filled approximately 5 cm deep with corncob bedding material evenly distributed across the cage. During the habituation phase, mice were introduced to the cage without any marbles and allowed to explore for 30 min. Twelve glass marbles (1.4 cm in diameter, plain dark glass) were then spaced evenly in a 3 × 4 grid on the bedding surface. During the testing phase, each mouse was placed in the cage and allowed to explore for 30 min. The number of marbles buried with bedding up to two-thirds of their depth was counted. All analyses were performed blind.

#### NBT

The NBT was conducted as described in previous reports (63). Mice were singly housed with corncob bedding and no environmental enrichment items, with ad libitum access to food and water. The nest, a small piece of pressed cotton, was used for nest building. Each mouse was acclimated for 24 h before the test. One piece of nestlet was placed in the corner of each cage at 10 AM. The next morning at 10 AM, the nests were scored. The nest scoring rubric was adapted from previous methods (64). A score of 0 was given if the nesting material was untouched, while scores of one to five were based on the quality and structure of the nest. The experimenter scored all four quadrants of the nest, and the scores were averaged for a total nest score.

#### TST

Depression-like behavior was examined using the TST, as previously described (65). The test apparatus consisted of two white acrylic walls (20 × 40 × 60 cm^3^) with one open wall for video recording. Four animals could be tested simultaneously. Each mouse was suspended by the tail 60 cm above the floor using adhesive tape placed less than 1 cm from the tip of the tail. Behavior was recorded for 6 min and later analyzed to determine the total duration of immobility. Immobility was defined as the period when the animals stopped struggling for at least 1 s. Data acquisition and analysis were performed using video tracking software (Med Associates).

#### FST

The FST was also used to examine depression-like behavior. The apparatus consisted of four Plexiglas cylinders (20 cm height × 10 cm diameter) filled with water at 22 to 24 °C to a depth of 7.5 cm^65^. Mice were placed in the cylinders for 6 min and video recorded. Behavior was analyzed to determine the total duration of immobility, defined as the period when the animals stopped struggling for at least 1 s. Data acquisition and analysis were performed using video tracking software (Med Associates).

### Cell and culture condition

Primary mouse astrocytes were isolated as previously described (29). Briefly, after removing the meninges, the cortical tissue from neonatal C57BL/6 mice (P0-P3) was mechanically triturated and digested with 0.25% (w/v) trypsin for 8 to 10 min. The mixed cortical cells were then added to Dulbecco’s Modified Eagle’s medium (Invitrogen) supplemented with 10% (v/v) heat-inactivated fetal bovine serum (Gibco), centrifuged, and the supernatant was discarded. The cells were resuspended in Dulbecco’s Modified Eagle’s medium with 10% fetal bovine serum, streptomycin (100 μg/ml), and penicillin (100 U/ml), passed through a 70-μm nylon mesh cell strainer, and plated on poly-L-lysine (PLL, 0.05 mg/ml, Sigma) precoated 75 cm^2^ flasks. Cultures were maintained at 37 °C in a humidified atmosphere with 5% CO_2_, and the medium was replaced every 3 to 4 days. Once confluent (10–14 days), astrocytes were isolated from mixed glial cultures by shaking the flasks at 260 rpm at 37 °C for 2 h. The supernatant containing microglia was aspirated, and the remaining cells were incubated with a trypsin solution (0.05% trypsin and 1 mM EDTA in Hank’s Balanced Salt Solution) at 37 °C for 15 min to eliminate oligodendrocytes. The remaining cells were digested with 0.05% trypsin (1 mM EDTA) and passaged for experiments. Astrocyte purity was assessed by immunofluorescent staining using a GFAP antibody (Abcam; Cat# ab178846, RRID: AB_2636859), and only cultures with over 90% purity were used for the study.

### Cell Counting Kit-8 assay

Cell viability was measured using the Cell Counting Kit-8 (Dojindo). Primary mouse astrocytes were seeded on 96-well plates and treated with various concentrations of DBZ (0.5 μM, 1 μM, 5 μM, 10 μM). Cells treated with 0.1% (v/v) DMSO served as the vehicle control. After 48 h of incubation, 10 μl of Cell Counting Kit-8 reagent was added to each well, and the plate was incubated at 37 °C for 4 h. Absorbance was measured at 450 nm using a SpectraMAX M3 microplate reader (Molecular Devices).

### Immunofluorescent staining

After behavioral tests, mice were anesthetized with 5% pentobarbital sodium and perfused transcardially with ice-cold saline followed by 4% paraformaldehyde in 0.1 M PBS (pH 7.4). The brains were postfixed in the same fixative for 12 h and dehydrated using a sucrose gradient (20% and 30% sucrose in 0.1 M PBS) at 4 °C overnight. Brain sections (20 μm thick) of the ACC, hippocampus, and amygdala were cut using a cryostat (Leica Microsystems). Sections were washed with 0.1 M PBS, permeabilized with 0.3% Triton X-100, and blocked with 10% bovine serum albumin in 0.1% Triton X-100 PBS for 2 h at room temperature. They were then incubated with GFAP antibodies (1:1500) overnight at 4 °C. Negative controls were incubated without primary antibodies. After rinsing three times with 0.1% Triton X-100 PBS, sections were incubated with goat anti-mouse IgG and CY3 (Servicebio) at room temperature for 2 h, followed by washing for 4 × 10 min in 0.1% Triton X-100 PBS. DAPI (Servicebio) was used to stain nuclei. Sections were mounted with medium containing antifade agents (Servicebio). Immunofluorescence images were acquired using a laser-scanning confocal microscope (FV1000, Olympus) equipped with FV10-ASW 4.0 VIEW software (Olympus). Four fields per section from five sections per animal were analyzed by a blinded observer, and the mean number of GFAP+ cells was calculated for each animal (n = 5 per group).

### ELISA for inflammatory cytokines

The levels of inflammatory cytokines in blood samples, brain tissues, and cell culture media were measured using ELISA kits (R&D Systems). Briefly, cells were cultured in 96-well plates and incubated with test compounds in serum-free medium for specified times. Media were collected for analysis. Brain tissues from the ACC, hippocampus, and amygdala were collected after behavioral tests, and blood samples were obtained *via* eyeball extraction. Supernatants were analyzed for TNF-α, IL-6, and IL-1β levels according to the manufacturer’s instructions. Absorbance was measured at 450 nm using a microplate reader (Molecular Devices).

### Oxidative stress ROS, MDA, and SOD measurement

ROS levels were measured using a DCFH-DA assay kit, MDA levels using a TBA assay kit, and SOD activity using a WST-1 assay kit, all from Nanjing Jiancheng Bioengineering Institute. Intracellular ROS was quantified by the oxidation of DCFH-DA to DCF. Briefly, brain samples from the ACC, hippocampus, and amygdala were made into single-cell suspensions, and primary astrocytes in 96-well plates were incubated with DCFH-DA (10 μM) for 30 min at 37 °C in the dark. Fluorescence was measured at excitation/emission wavelengths of 485/535 nm using a fluorescence microplate reader (Spectra Max Gemini EM, Molecular Devices), and images were captured using a laser-scanning confocal microscope (FV1000, Olympus) by blinded investigators.

MDA was measured *via* the formation of MDA-TBA adducts, which absorb at 532 nm. Brain samples were homogenized to 10%, and supernatants were collected from treated primary astrocytes. Test samples (100 μl) were processed according to the manufacturer’s instructions, incubated in a water bath at 95 °C for 40 min, cooled, centrifuged at 4000 rpm for 40 min, and the absorbance of supernatants was measured at 532 nm.

SOD activity was assessed using WST-1, which forms a water-soluble formazan dye upon reduction. Briefly, 20 μl of sample (tissue homogenate, cell supernatant, and plasma) and 20 μl of enzyme working solution were added to the sample well, followed by 200 μl of WST working solution, and incubated at 37 °C for 20 min. Absorbance was measured at 450 nm using a microplate reader, and SOD activity was calculated using the equation: SOD activity (U/ml) = (A_blank1_ - A_sample_)/(A_blank1_ - A_blank2_) × 40.

### Western blot analysis

Brain tissues were homogenized in ice-cold RIPA lysis buffer (50 mM Tris-Cl, pH 7.4, 150 mM NaCl, 0.1% SDS, 1% sodium deoxycholate, and 1% Triton X-100) with protease and phosphatase inhibitors (Abcam; Cat# ab201120). Protein concentration was determined using a BCA assay kit (Beyotime Biotech). Proteins were separated by SDS-PAGE, transferred to PVDF membranes (GE Healthcare), blocked in TBST with 5% BSA or nonfat dry milk, and incubated overnight at 4 °C with primary antibodies, followed by HRP-conjugated secondary antibodies for 1 h at room temperature. Immunoreactive bands were detected with ECL-Plus reagents (GE Healthcare). The following antibodies were used: rabbit anti-Jak2 (CST, UK; Cat# 3230, RRID: AB_2128522), rabbit anti-phospho-Jak2 (Tyr1007/1008) (CST, UK; Cat# 3771, RRID: AB_330403), rabbit anti-Stat3 (CST, UK; Cat# 12640S, RRID: AB_2629499), mouse anti-phospho-Stat3 (Tyr705) (CST, UK; Cat# 4113, RRID: AB_2198588), rabbit anti-β-actin (CST, UK; Cat# 4970, RRID: AB_2223172), HRP-linked goat anti-rabbit IgG (CST, UK; Cat# 7074, RRID: AB_2099233), and HRP-linked horse anti-mouse IgG (CST, UK; Cat# 7076, RRID: AB_330924). Band densities were analyzed using ImageJ software (NIH), and phosphorylated/total protein or target protein/β-actin ratios were calculated. Data collection was performed by blinded investigators. For each immunoblot experiment, the most appropriate loading control was selected based on preliminary optimization. Specifically, GAPDH was used when its expression exhibited minimal variation and matched the molecular weight range of the target protein, while beta-actin was used in experiments where it provided more reliable normalization.

### Quantitative RT-PCR

Total RNA was extracted from brain tissues using Trizol reagent (Invitrogen) and reverse transcribed using the PrimeScript RT reagent Kit (TaKaRa). RNA integrity was confirmed by electrophoresis, and RNA quantification was performed spectrophotometrically at 260 nm. Relative mRNA levels were determined using SYBR Fast qPCR Mix (TaKaRa) and a real-time PCR system (iCycler iQ, Bio-Rad). Data were normalized to GAPDH, and fold change was calculated using the 2^ˆ^-ΔΔCt method. The primers used were as follows: **GAPDH** (sense, 5′-CGACTTCAACAGCAACTCCCACTCTTCC-3′, antisense, 5′-TGGGTGGTCCA GGGTTTCTTACTCCTT-3′); **TNF-α** (sense, 5′-ATGAGCACAGAAAGCATGATC-3′, antisense 5′-TACAGGCTTGTCACTCGAATT-3′); **IL-6** (sense, 5′-ACTTCACAAGTCGGAGGCTT-3′, antisense, 5′-TGCAAGTGCATCATCGTTGT-3′); **IL-1β** (sense, 5′-ATGGCAACTGTTCCTGAACTCAACT-3′, antisense, 5′-TTTCCTTTCTTAGATATGGACAGGAC-3′).

### Network pharmacology

We explored the possible pathways of DBZ action through network pharmacology, following previous studies (66, 67). The main elements included are prediction of potential targets of DBZ, inflammatory pain genes, protein-protein interactions network of DBZ targets and inflammatory pain, gene ontology analysis, and Kyoto Encyclopedia of Genes and Genomes pathway enrichment analysis.

### Molecular docking analysis

Docking analyses of DBZ with JAK2 and STAT3 were performed using the Glide module of Maestro 11.9 (68, 69). Protein structures were downloaded from the Protein Data Bank (http://www.rcsb.org) and prepared using the Protein Preparation Wizard Workflow in the Schrödinger suite. Hydrogen atoms were added, bond orders assigned, and unnecessary water molecules removed. H-bonds were optimized, and restrained minimization performed with heavy atoms converged to 0.3 Å RMSD. DBZ and the original crystal ligands were prepared using the Ligand Preparation Application in the Schrödinger suite. The Receptor Grid Generation Workflow defined a grid around the bound cocrystallized ligand for docking DBZ in the ligand-binding site. Extra Precision (XP) mode was used for docking analyses. The ligand interaction tool was used to view interaction diagrams of ligands with residues at the active site of the target protein.

### Statistical analysis

Results are expressed as mean ± standard error of the mean. Statistical analysis of multiple groups was performed using one-way analysis of variance with *post hoc* tests in Microsoft Excel and Prism (GraphPad). A *p*-value < 0.05 was considered statistically significant. In our study, we conducted an *a priori* power analysis using software G∗Power, basing our calculations on effect sizes observed in preliminary experiments and similar studies in the literature. For *in vitro* assays, where variability was relatively low, a sample size of n = 3 was sufficient to achieve a power of 80% at an α level of 0.05. However, recognizing the higher biological variability in *in vivo* experiments and certain molecular assays, we increased the number of replicates to n = 8 when feasible. This approach allowed us to balance statistical rigor with practical experimental constraints, ensuring that our findings are robust and reproducible.

## Data availability

All data described in the article are contained in the main test.

## Conflict of interest

The authors declare they have no conflicts of interest with the contents of this article.
